# Incremental Prognostic Value of the Haemoglobin-Albumin-Lymphocyte-Platelet Score and B-Type Natriuretic Peptide for 30-Day Mortality After Elective On-Pump Coronary Artery Bypass Grafting

**DOI:** 10.1093/icvts/ivag190

**Published:** 2026-06-30

**Authors:** Hafize Yalınız, Onur Benli

**Affiliations:** Department of Cardiovascular Surgery, Cukurova University Faculty of Medicine, 01433 Adana, Turkey; Department of Cardiovascular Surgery, Cukurova University Faculty of Medicine, 01433 Adana, Turkey

**Keywords:** coronary artery bypass grafting, EuroSCORE II, inflammation, biomarkers

## Abstract

**Objectives:**

This study evaluated whether preoperative haemoglobin-albumin-lymphocyte-platelet (HALP) score and B-type natriuretic peptide (BNP) were associated with 30-day mortality after elective on-pump coronary artery bypass grafting (CABG) and whether they improved prognostic assessment beyond European System for Cardiac Operative Risk Evaluation II (EuroSCORE II).

**Methods:**

Among 492 eligible patients, 466 with complete data on 30-day mortality and the prespecified primary predictors constituted the primary analytic cohort. Associations were examined using Firth penalized logistic regression, and internal validation was performed with bootstrap resampling.

**Results:**

Twenty-one patients (4.5%) died within 30 days. Lower HALP and higher BNP were independently associated with mortality after adjustment for EuroSCORE II. The optimism-corrected area under the curve improved from 0.879 for EuroSCORE II alone to 0.974 after the addition of HALP and to 0.979 after further addition of BNP.

**Conclusions:**

Lower HALP and higher BNP were associated with 30-day mortality after elective on-pump CABG and may provide incremental prognostic information beyond EuroSCORE II. These findings should be considered exploratory.

## Graphical Abstract

**Figure:**
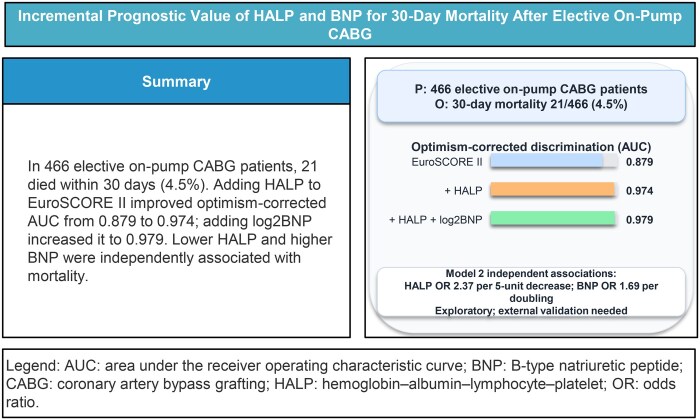

## INTRODUCTION

Coronary artery bypass grafting (CABG) remains a standard revascularization strategy for patients with multivessel coronary artery disease, particularly when coronary anatomy is complex or percutaneous intervention is less suitable.^1^ Despite advances in surgical technique and perioperative care, early postoperative mortality remains clinically relevant. Accurate preoperative risk assessment therefore remains important for patient counselling, perioperative planning, and outcome evaluation.^2^^,^^3^

Established models such as European System for Cardiac Operative Risk Evaluation II (EuroSCORE II) are widely used for operative risk stratification in cardiac surgery.^4^^,^^5^ However, they are based largely on demographic, clinical, and procedural variables and may not fully capture biological processes that influence perioperative vulnerability, including inflammation, nutritional reserve, immune competence, and myocardial stress.^6^^,^^7^

The haemoglobin-albumin-lymphocyte-platelet (HALP) score integrates haematologic, nutritional, and immune-inflammatory parameters and may reflect physiological reserve before surgery.^8–10^ B-type natriuretic peptide (BNP), a marker of ventricular wall stress and volume overload, has been linked to adverse outcomes after cardiac surgery.^11^ Other inflammatory indices, including the systemic immune-inflammation index (SII) and pan-immune-inflammation value (PIV), may capture related but partly overlapping biological domains.^12^^,^^13^ B-type natriuretic peptide may provide complementary prognostic information by reflecting myocardial stress and haemodynamic burden.^11^^,^^14^

The prognostic roles of HALP, BNP, SII, and PIV in elective on-pump CABG, particularly their incremental value beyond EuroSCORE II for 30-day mortality, remain insufficiently defined. Therefore, we examined the association of these preoperative biomarkers with 30-day mortality and evaluated whether HALP and BNP improved risk assessment beyond EuroSCORE II. Given the limited number of events, this was designed as an exploratory analysis rather than a definitive prediction study.

## METHODS

### Study design and population

This retrospective single-centre cohort study included consecutive adult patients who underwent elective isolated on-pump CABG at the Department of Cardiovascular Surgery, Cukurova University, between January 2010 and December 2019. Patients undergoing concomitant valvular or aortic procedures, off-pump CABG, emergency surgery, active infection, or dialysis-dependent end-stage renal disease were excluded. Of 721 screened cardiac surgery patients, 492 met eligibility criteria. The primary analytic cohort consisted of 466 patients with complete 30-day mortality status and complete data for the prespecified primary predictors. The primary end-point was 30-day all-cause mortality, determined from hospital records and the national death registry.

All patients underwent preoperative coronary angiographic evaluation and were referred for CABG after institutional cardiology-cardiovascular surgery board assessment. However, detailed coronary anatomical variables such as SYNTAX score, left main coronary artery disease, prior percutaneous coronary intervention (PCI), and prior myocardial infarction were not uniformly available in the surgical research database and were therefore not included in the analysis.

Demographic and clinical data were retrieved from the institutional electronic medical record system. Recorded variables included age, sex, chronic obstructive pulmonary disease, diabetes mellitus, chronic kidney disease, extracardiac arteriopathy, previous cardiac surgery, pulmonary artery pressure ≥40 mmHg, left ventricular ejection fraction, New York Heart Association (NYHA) class, serum creatinine, EuroSCORE II, cross-clamp time, cardiopulmonary bypass (CPB) time, and distal graft number.

EuroSCORE II was analysed primarily as a continuous variable. For descriptive purposes, patients were also grouped as low risk (0-2), intermediate risk (3-5), and high risk (≥6).

Preoperative laboratory values were obtained within 72 hours before surgery. Haemoglobin-albumin-lymphocyte-platelet score was calculated as haemoglobin × albumin × lymphocyte count/platelet count, SII as neutrophil count × platelet count/lymphocyte count, and PIV as neutrophil count × platelet count × monocyte count/lymphocyte count. B-type natriuretic peptide was measured using standard institutional immunoassays. Haemoglobin-albumin-lymphocyte-platelet score and BNP were prespecified as the primary biomarkers for incremental modelling beyond EuroSCORE II, whereas SII and PIV were evaluated as secondary exploratory biomarkers.

The normality of continuous variables was assessed using the Shapiro–Wilk test and visual inspection of histograms and Q-Q plots. Continuous variables are presented as mean ± SD or median (IQR), and categorical variables as counts and percentages. Group comparisons used the Student’s t-test or Mann–Whitney *U* test for continuous variables and the chi-square test or Fisher’s exact test for categorical variables, as appropriate.

Because only 21 deaths occurred, parsimonious penalized models were prespecified to reduce small-sample bias and overfitting. Firth penalized logistic regression was chosen because it provides more stable estimates than conventional logistic regression when the number of events is small. B-type natriuretic peptide, SII, and PIV were log2-transformed because their distributions were right-skewed; therefore, the odds ratios (ORs) for these variables represent the change in the odds of 30-day mortality for each doubling of the biomarker value. Bootstrap internal validation was used to estimate potential optimism in model performance. Calibration measures and the Brier score were reported to assess whether predicted mortality risks were close to the observed mortality rates. Univariable and multivariable associations with 30-day mortality were examined using Firth penalized logistic regression, with EuroSCORE II retained as the baseline clinical model. Age was not entered separately because it is already included in EuroSCORE II. EuroSCORE II was used as the composite operative risk adjustment variable; therefore, individual operative risk factors were not entered separately into the multivariable models.

Haemoglobin-albumin-lymphocyte-platelet score was analysed continuously and scaled per 5-unit decrease. B-type natriuretic peptide, SII, and PIV were right-skewed and were therefore log2-transformed; ORs are reported per doubling. The primary multivariable models were limited to EuroSCORE II + HALP and EuroSCORE II + HALP + log2BNP. Systemic immune-inflammation index and PIV were evaluated in separate secondary models. Discrimination was assessed by the area under the receiver operating characteristic curve (AUC), and calibration by calibration-in-the-large, calibration slope, and Brier score. Internal validation used 1000 bootstrap resamples. Additional analyses compared included and excluded patients, applied inverse-probability-of-inclusion weighting, and performed tipping-point sensitivity analyses. No imputation was performed.

All statistical analyses were performed using R statistical software.

### Ethical approval

The study protocol was approved by the Ethics Committee of Cukurova University Faculty of Medicine (May 15, 2020; approval no. 99; decision no. 14). Because of the retrospective design and use of anonymized clinical data, the requirement for written informed consent was waived. The study was conducted in accordance with the Declaration of Helsinki. No biological material was collected, stored, or used for biobank purposes, and no data or biological material were stored for multiple or indefinite future research use under the scope of the WMA Declaration of Taipei.

## RESULTS

**Figure 1.**
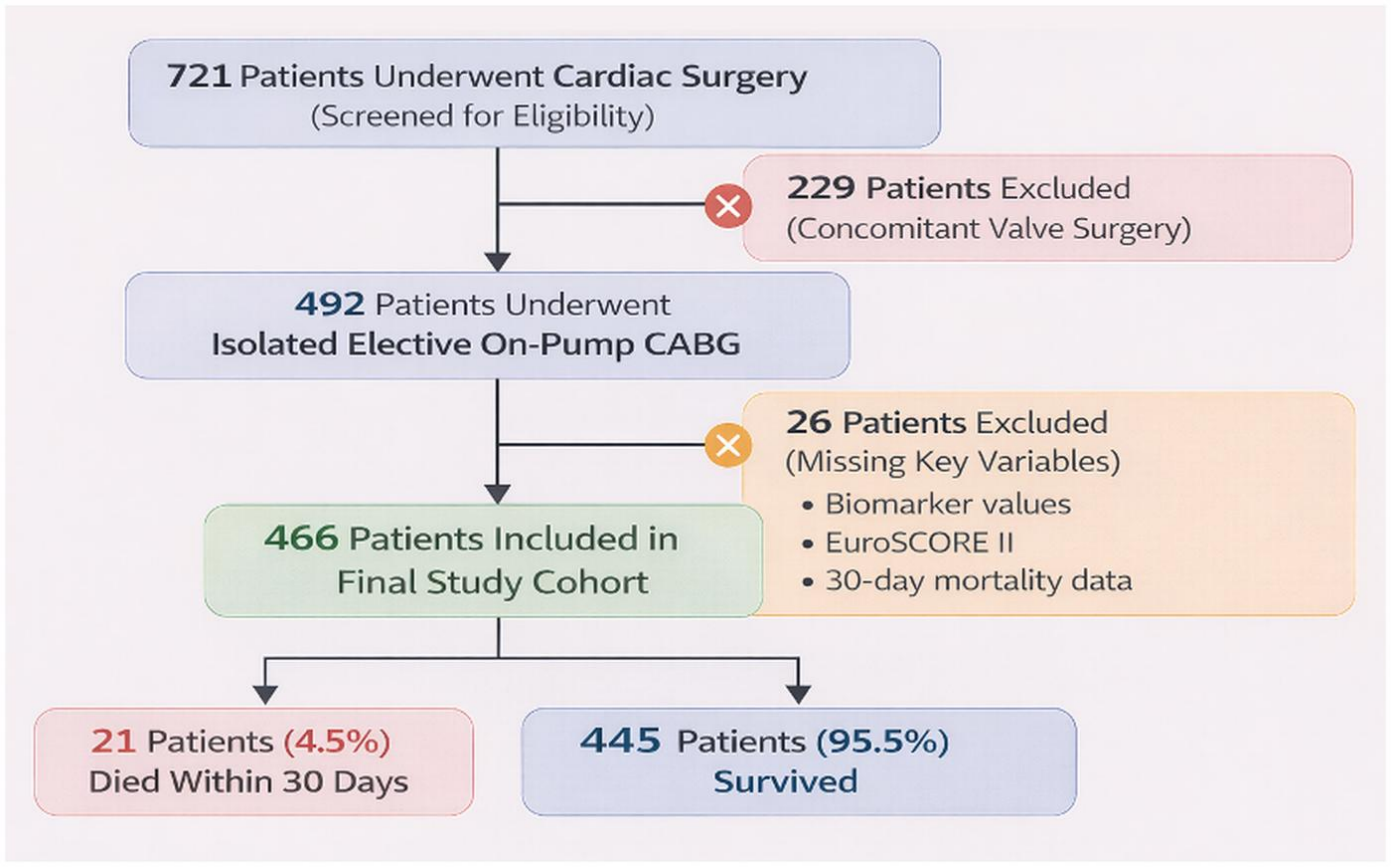
Flow Diagram of Cohort Derivation. A total of 721 patients who underwent cardiac surgery during the study period were screened. After exclusion of 229 patients according to the study protocol, 492 patients remained in the eligible isolated elective on-pump CABG cohort. Of these, 26 otherwise eligible patients were not included in the primary analytic cohort because 30-day mortality status and/or prespecified primary predictor data were unavailable. The final analytic cohort consisted of 466 patients, including 445 survivors and 21 non-survivors at 30 days. Abbreviation: EuroSCORE II, European System for Cardiac Operative Risk Evaluation II.

Of 721 screened patients, 492 constituted the eligible isolated elective on-pump CABG cohort and 466 formed the primary analytic cohort; 445 patients (95.5%) survived and 21 (4.5%) died within 30 days (**Figure 1**). Among the 21 patients who died within 30 days, the recorded dominant causes of death were low cardiac output syndrome leading to multiorgan failure in 12 patients (57.1%), malignant arrhythmia or sudden cardiac death in 3 patients (14.3%), major bleeding or haemorrhagic complications in 3 patients (14.3%), and unknown or not clearly documented causes in 3 patients (14.3%). Missingness was clustered rather than diffuse: 455 patients (92.5%) had complete data for all listed variables, 11 (2.2%) had isolated missing creatinine, and the remaining 26 accounted for all missing 30-day outcome data and most additional missingness in EuroSCORE II, NYHA class, biomarker variables, and cross-clamp time (**Tables S1**  **and**  **S2**).

Compared with included patients, the 26 excluded but otherwise eligible patients were not materially older but appeared to have a less favourable operative and inflammatory profile, including longer CPB times, lower left internal mammary artery use, and higher available PIV and SII values (**Table 1**).

**Table 1.** Included vs Excluded Eligible Patients (*n* = 492)

Characteristic	Included in primary analytic cohort (*n* = 466)	Excluded from primary analytic cohort (*n* = 26)	SMD	*P* value
Age, years	61.0 (54.0-69.0)	59.5 (57.0-65.8)	0.073	.828
Male sex	333 (71.5%)	14 (53.8%)	0.364	.075
CPB time, min	108 (85-130)	132 (106-154)	0.689	<.001
Distal graft number	3 (2-3)	3 (3-3)	0.455	.037
LIMA use	232 (49.8%)	7 (26.9%)	0.470	.026
PIV^a^	374.7 (289.4-539.5)	565.2 (435.6-1141.7)	0.662	.018
SII^b^	731.2 (556.9-1021.0)	936.8 (817.0-1273.5)	0.613	.043

*P* values were calculated using the Mann–Whitney *U* test for continuous variables and Fisher’s exact test for categorical variables.

a PIV was available in 466 included patients and 9 excluded patients.

b SII was available in 466 included patients and 8 excluded patients.

Abbreviations: CPB, cardiopulmonary bypass; LIMA, left internal mammary artery; PIV, pan-immune-inflammation value; SII, systemic immune-inflammation index; SMD, standardized mean difference.

Non-survivors were older than survivors (70.0 [67.0-76.0] vs 61.0 [53.0-68.0] years, *P* < .001) and had higher rates of diabetes mellitus, chronic kidney disease, extracardiac arteriopathy, pulmonary artery pressure ≥40 mmHg, and NYHA class III symptoms. They also had higher creatinine and EuroSCORE II values, longer CPB times, and lower left internal mammary artery use, whereas cross-clamp time and distal graft number did not differ significantly. Non-survivors also had a less favourable left ventricular ejection fraction profile than survivors, with a higher proportion of poor or moderately reduced ejection fraction categories and a lower proportion of preserved ejection fraction (*P* < .001) (**Table 2**).

**Table 2.** Survivors vs Non-survivors within the Analytic Cohort (*n* = 466)

Variable	Survivors (*n* = 445)	Non-survivors (*n* = 21)	*P* value
Age, years	61.0 (53.0-68.0)	70.0 (67.0-76.0)	<.001
Male sex	322 (72.4%)	11 (52.4%)	.080
COPD	45 (10.1%)	4 (19.0%)	.261
Diabetes mellitus	152 (34.2%)	15 (71.4%)	<.001
Chronic kidney disease	9 (2.0%)	5 (23.8%)	<.001
Extracardiac arteriopathy	58 (13.0%)	10 (47.6%)	<.001
Pulmonary artery pressure ≥40 mmHg	0 (0.0%)	9 (42.9%)	<.001
NYHA class III	4 (0.9%)	2 (9.5%)	.026
Serum creatinine, mg/dL^a^	0.88 (0.75-1.13)	1.08 (0.90-1.17)	.036
EuroSCORE II	2.0 (1.0-4.0)	6.0 (6.0-7.0)	<.001
Cross-clamp time, min	60.0 (50.0-80.0)	65.0 (60.0-80.0)	.070
CPB time, min	105.0 (85.0-130.0)	130.0 (120.0-160.0)	<.001
LIMA use	227 (51.0%)	5 (23.8%)	.023
Distal graft number^b^	3.0 (2.0-3.0)	3.0 (3.0-3.0)	.073
HALP	35.1 (30.1-44.8)	17.3 (10.6-19.3)	<.001
BNP, pg/mL	250.0 (160.0-600.0)	2400.0 (600.0-4000.0)	<.001
PIV	363.8 (286.7-523.3)	608.9 (496.1-705.6)	<.001
SII	725.8 (553.2-1004.8)	1144.5 (784.0-1587.2)	<.001
Left ventricular ejection fraction category			<.001
Poor LVEF	8 (1.8%)	3 (14.3%)	
Moderate LVEF	191 (42.9%)	17 (81.0%)	
Preserved LVEF	246 (55.3%)	1 (4.8%)	

*P* values were calculated using the Mann–Whitney *U* test for continuous variables and Fisher’s exact test for categorical variables.

a Serum creatinine was available in 434 survivors and 21 non-survivors.

b Distal graft number was available in 444 survivors and 21 non-survivors.

Abbreviations: BNP, B-type natriuretic peptide; CKD, chronic kidney disease; COPD, chronic obstructive pulmonary disease; CPB, cardiopulmonary bypass; EuroSCORE II, European System for Cardiac Operative Risk Evaluation II; HALP, haemoglobin-albumin-lymphocyte-platelet score; LIMA, left internal mammary artery; LVEF, left ventricular ejection fraction; NYHA, New York Heart Association; PIV, pan-immune-inflammation value; SII, systemic immune-inflammation index.

Biomarker distributions also differed substantially between groups. Haemoglobin-albumin-lymphocyte-platelet score was lower in non-survivors (17.3 [10.6-19.3] vs 35.1 [30.1-44.8], *P* < .001), whereas BNP (2400 [600-4000] vs 250 [160-600] pg/mL, *P* < .001), PIV (608.9 [496.1-705.6] vs 363.8 [286.7-523.3], *P* < .001), and SII (1144.5 [784.0-1587.2] vs 725.8 [553.2-1004.8], *P* < .001) were higher in non-survivors. No deaths occurred in the low-risk EuroSCORE II group, whereas 6 occurred in the intermediate-risk group and 15 in the high-risk group.

In univariable Firth logistic regression, higher EuroSCORE II, log2BNP, log2SII, and log2PIV values were associated with increased odds of 30-day mortality, whereas higher HALP was associated with lower risk. Haemoglobin-albumin-lymphocyte-platelet score showed the strongest biomarker association (OR 2.42 per 5-unit decrease, 95% CI 1.83-3.20; *P* < .001), and EuroSCORE II was also strongly associated with mortality (OR 1.87 per 1-unit increase, 95% CI 1.49-2.35; *P* < .001) (**Table S3**).

In the prespecified multivariable model including EuroSCORE II and HALP, both variables remained independently associated with 30-day mortality. In the secondary model that additionally included log2BNP, EuroSCORE II, HALP, and log2BNP all remained significant (**Table 3**).

**Table 3.** Primary Multivariable Firth Penalized Logistic Regression Models for 30-Day Mortality in the Analytic Cohort (*n* = 466)

Variable	Model 1: EuroSCORE II + HALP OR (95% CI)	*P* value	Model 2: EuroSCORE II + HALP + log2BNP OR (95% CI)	*P* value
EuroSCORE II (per 1-unit increase)	1.74 (1.33-2.27)	<.001	1.61 (1.22-2.14)	<.001
HALP (per 5-unit decrease)	2.23 (1.66-3.00)	<.001	2.37 (1.67-3.37)	<.001
log2BNP (per doubling)	—	—	1.69 (1.27-2.25)	<.001

Odds ratios were estimated using multivariable Firth penalized logistic regression with 30-day mortality as the dependent variable. Haemoglobin-albumin-lymphocyte-platelet score was scaled per 5-unit decrease to improve clinical interpretability. BNP was log2-transformed; the corresponding odds ratio therefore represents the relative increase in the odds of 30-day mortality per doubling of BNP. Model 1 was the prespecified primary model. Model 2 was the secondary model evaluating the incremental prognostic contribution of BNP beyond EuroSCORE II and HALP.

Abbreviations: BNP, B-type natriuretic peptide; CI, confidence interval; EuroSCORE II, European System for Cardiac Operative Risk Evaluation II; HALP, haemoglobin-albumin-lymphocyte-and platelet score; OR, odds ratio.

**Figure 2.**
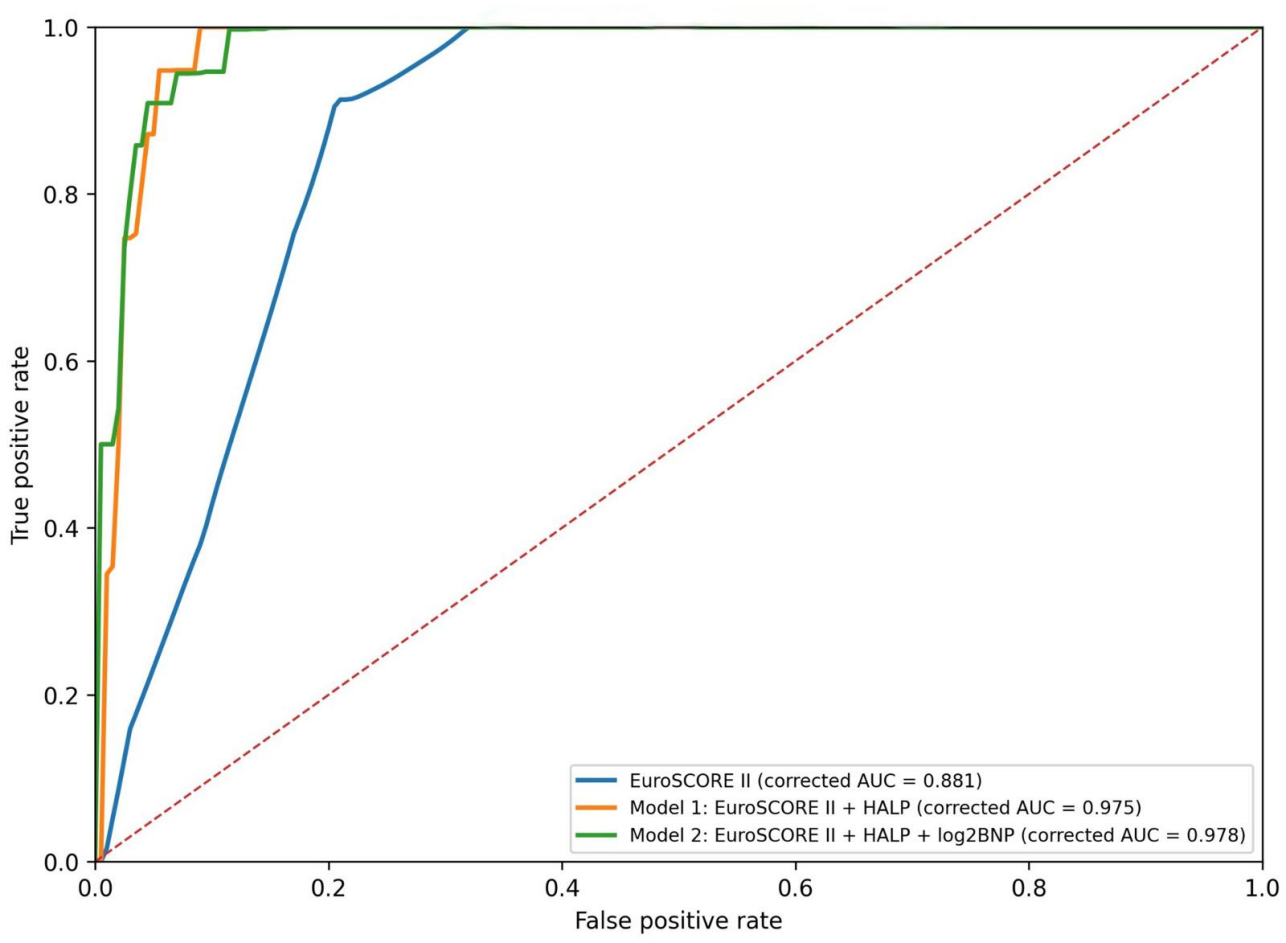
Bootstrap-Corrected ROC Curves. Bootstrap optimism-corrected receiver operating characteristic curves are shown for EuroSCORE II alone, model 1 (EuroSCORE II + HALP), and model 2 (EuroSCORE II + HALP + log2BNP) in the analytic cohort. Discrimination improved after addition of HALP and improved further after addition of log2BNP. Corrected AUC values are displayed in the legend. Abbreviations: AUC, area under the receiver operating characteristic curve; EuroSCORE II, European System for Cardiac Operative Risk Evaluation II; HALP, haemoglobin-albumin-lymphocyte-platelet score.

Discrimination improved after the sequential addition of HALP and BNP to EuroSCORE II. Apparent AUC increased from 0.881 for EuroSCORE II alone to 0.976 for EuroSCORE II + HALP and to 0.980 after further addition of log2BNP; optimism-corrected AUCs were 0.879, 0.974, and 0.979, respectively (**Table 4**; **Figure 2**).

**Table 4.** Model Performance and Internal Validation in the Analytic Cohort (*n* = 466)

Model	Apparent AUC	Optimism-corrected AUC^a^	Optimism-corrected calibration slope^a^	Optimism-corrected Brier score^a^	Incremental LR test^b^
European System for Cardiac Operative Risk Evaluation II	0.881	0.879	1.009	0.040	Reference
Model 1: European System for Cardiac Operative Risk Evaluation II + haemoglobin-albumin-lymphocyte-platelet score	0.976	0.974	0.952	0.030	<0.001 vs European System for Cardiac Operative Risk Evaluation II
Model 2: European System for Cardiac Operative Risk Evaluation II + haemoglobin-albumin-lymphocyte-platelet score + log2-transformed B-type natriuretic peptide	0.980	0.979	0.986	0.024	<0.001 vs Model 1

a Optimism-corrected estimates were obtained by bootstrap internal validation.

b Incremental likelihood-ratio (LR) tests compared model 1 with the European System for Cardiac Operative Risk Evaluation II alone and model 2 with model 1.

Abbreviations: AUC, area under the receiver operating characteristic curve; LR, likelihood ratio.

**Figure 3.**
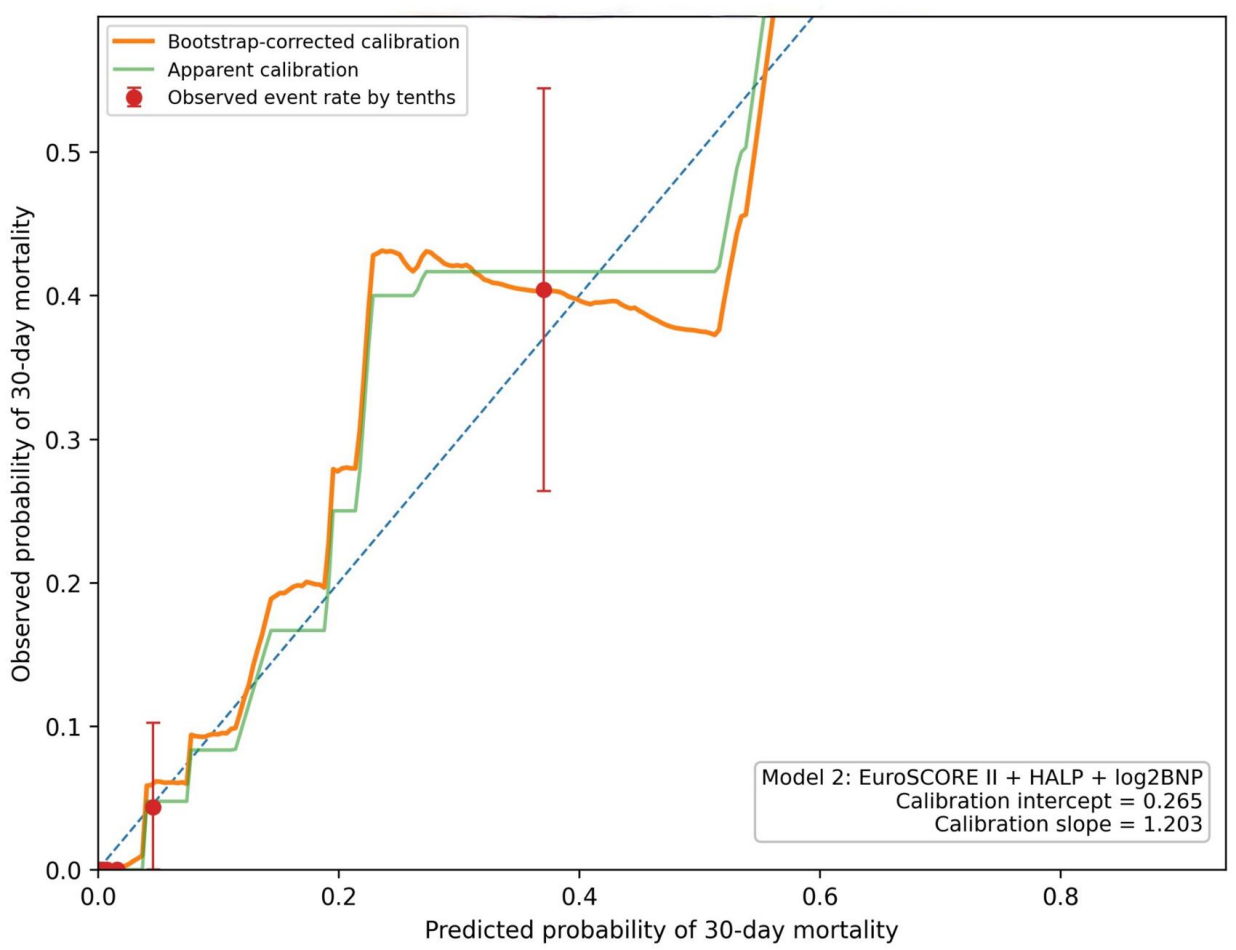
Apparent Calibration Plot for Model 2. The figure shows apparent calibration for model 2 (European System for Cardiac Operative Risk Evaluation II + haemoglobin-albumin-lymphocyte-platelet score + log2-transformed B-type natriuretic peptide) in the analytic cohort. The dashed diagonal line represents perfect calibration. Points indicate observed 30-day mortality by tenths of predicted risk, with approximate 95% CIs. The solid curve represents the apparent calibration function. Abbreviations: EuroSCORE II, European System for Cardiac Operative Risk Evaluation II; HALP, haemoglobin-albumin-lymphocyte-platelet score.

Calibration remained acceptable across models. Optimism-corrected calibration slopes were 1.009 for EuroSCORE II alone, 0.952 for EuroSCORE II + HALP, and 0.986 for EuroSCORE II + HALP + log2BNP, with corresponding optimism-corrected Brier scores of 0.040, 0.030, and 0.024. Incremental likelihood-ratio testing showed that HALP improved fit beyond EuroSCORE II and that BNP provided additional improvement beyond the HALP-augmented model (**Table 4**; **Figure 3**).

Spline analyses showed progressively higher adjusted odds of 30-day mortality at lower HALP values and at higher log2BNP values across the observed ranges (**Figures S1**  **and**  **S2**). Decision curve analysis indicated greater net benefit for both biomarker-augmented models than for EuroSCORE II alone, with the EuroSCORE II + HALP + log2BNP model showing the highest overall net benefit (**Figure S3**).

Sensitivity analyses supported the primary findings. In inverse-probability-of-inclusion weighted models, EuroSCORE II, lower HALP, and higher BNP remained materially associated with 30-day mortality. Tipping-point analyses suggested that overall mortality in the full eligible cohort would change only modestly under plausible assumptions about excluded patients. Systemic immune-inflammation index and PIV were associated with mortality when assessed separately beyond EuroSCORE II, but neither remained independently associated after addition of HALP. Ridge-penalized models yielded results similar to the Firth models (**Tables S4-S7**).

## DISCUSSION

In this single-centre retrospective cohort of elective on-pump CABG patients, lower HALP and higher BNP were associated with 30-day mortality and appeared to improve risk stratification beyond EuroSCORE II. These findings should be interpreted cautiously because only 21 deaths occurred and 26 otherwise eligible patients were excluded because of unavailable outcome and/or predictor data.

The observed overall 30-day mortality rate was 4.5%, but deaths were concentrated in patients with higher baseline risk: 71.4% occurred in the EuroSCORE II high-risk group and none occurred in the low-risk group. This pattern suggests that the cohort’s overall mortality largely reflected its underlying risk distribution rather than an unexplained excess event rate.

Our BNP findings are consistent with previous cardiac surgery literature linking elevated preoperative natriuretic peptide levels to low cardiac output, renal dysfunction, prolonged intensive care, and mortality.^15–17^ B-type natriuretic peptide likely contributes information not fully captured by conventional risk scores because it directly reflects myocardial stress and haemodynamic burden.

Although the addition of BNP to the EuroSCORE II + HALP model produced only a small further increase in AUC, BNP remained independently associated with 30-day mortality and improved model fit, Brier score, and decision-curve performance. Therefore, BNP may provide complementary information related to myocardial stress and haemodynamic burden. However, the modest gain in discrimination indicates that its incremental clinical value should be interpreted cautiously. B-type natriuretic peptide should not be viewed as a standalone decision-making marker, and its practical utility requires confirmation in externally validated cohorts.

Haemoglobin-albumin-lymphocyte-platelet score showed the strongest and most consistent biomarker association with mortality in this cohort. Because HALP combines anaemia, nutritional status, immune competence, and platelet-related inflammatory burden, it may summarize perioperative vulnerability more broadly than single-domain biomarkers. Although HALP has been studied mainly in oncology and only recently in CABG populations, our findings support its potential relevance in cardiac surgical risk assessment.^8–10^^,^^18^

Haemoglobin-albumin-lymphocyte-platelet score may be relevant in cardiac surgery because it integrates anaemia, nutritional reserve, immune status, and platelet-related inflammatory burden. Lower haemoglobin may reflect reduced oxygen-carrying capacity, hypoalbuminaemia may indicate malnutrition or systemic inflammation, lymphopenia may reflect impaired immune competence, and platelet count may be linked to thromboinflammatory activity. Therefore, a lower HALP should be interpreted as a composite surrogate of perioperative vulnerability and systemic illness burden rather than as evidence of a direct causal mechanism.^19^^,^^20^

The marked increase in AUC after adding HALP to EuroSCORE II should be interpreted cautiously. Despite similar apparent and optimism-corrected estimates, the limited number of deaths means that overfitting and cohort-specific discrimination cannot be excluded. External validation is required before this incremental performance can be considered clinically robust.

Systemic immune-inflammation index and PIV were also associated with mortality in univariable and EuroSCORE II-adjusted analyses, but their effects were attenuated after the addition of HALP. This suggests that these indices carry prognostic signal but may overlap biologically with the broader information captured by HALP.^21^^,^^22^ Accordingly, they should be viewed here as secondary exploratory markers rather than preferred components of the main model.

Clinically, these results do not justify a new standalone risk tool, but they suggest that routine preoperative biomarkers may refine risk assessment beyond EuroSCORE II and Society of Thoracic Surgeons (STS) alone.^14^^,^^23^ Because HALP and BNP are readily available in everyday practice, they may be practical candidates for future model refinement if confirmed externally.

These findings do not justify changes in surgical eligibility, timing, or operative strategy, and the model should not be used as a EuroSCORE-like bedside calculator without external validation. Haemoglobin-albumin-lymphocyte-platelet score and BNP may identify patients who warrant closer preoperative assessment or optimization of anaemia, nutritional status, inflammatory burden, or myocardial stress, but this study does not show that modifying these biomarkers improves postoperative outcomes.

This study has important limitations. It was retrospective, single-centre, and based on a limited number of deaths, which constrained model complexity and precision. Given the limited number of deaths and the use of internally validated models, these statistical results should be interpreted as supportive exploratory evidence rather than proof of a clinically ready prediction tool. Because the cohort was limited to elective isolated on-pump CABG, the findings cannot be directly generalized to off-pump CABG or to other cardiac surgical procedures; the prognostic value of HALP in these populations requires separate external validation.

Selection bias cannot be excluded because 26 otherwise eligible patients were not included in the primary analysis, although we addressed this with transparent missing-data reporting, inclusion-weighted analyses, and tipping-point sensitivity analyses. Residual confounding remains possible, particularly because detailed coronary anatomical variables such as SYNTAX score, left main coronary artery disease, prior PCI, and prior myocardial infarction were not uniformly available in the surgical research database. Although all patients underwent preoperative coronary angiographic assessment and institutional cardiology-cardiovascular surgery board evaluation before CABG, these anatomical variables could not be incorporated into the present analysis. In addition, standardized intraoperative bleeding volume, transfusion data, and intra-aortic balloon pump requirement were unavailable. B-type natriuretic peptide measurements may vary across laboratories, and composite indices such as HALP, SII, and PIV are not yet standardized for cardiac surgery. Nevertheless, the cohort was relatively homogeneous, all biomarkers were assessed within a single analytic framework, and model evaluation included calibration, internal validation, decision-curve analysis, and multiple sensitivity analyses.

## CONCLUSION

Lower HALP and higher BNP were associated with early mortality after elective on-pump CABG. In internally validated parsimonious models, these biomarkers appeared to provide incremental prognostic information beyond EuroSCORE II. However, the findings remain exploratory and hypothesis-generating.

## AUTHOR CONTRIBUTIONS

Hafize Yalınız and Onur Benli contributed to the conception and design of the study. Onur Benli collected the data. Hafize Yalınız and Onur Benli performed data interpretation. Onur Benli performed the statistical analysis and drafted the manuscript. Hafize Yalınız critically revised the manuscript for important intellectual content. Both authors approved the final version of the manuscript and agree to be accountable for all aspects of the work.

## Supplementary Material

ivag190_Supplementary_Data

## Data Availability

The data underlying this article are not publicly available due to institutional and patient confidentiality restrictions. De-identified data may be made available from the corresponding author on reasonable request, subject to approval by the institutional ethics committee and applicable institutional policies.
