# Supplementary material for: Incremental Prognostic Value of the Haemoglobin-Albumin-Lymphocyte-Platelet Score and B-Type Natriuretic Peptide for 30-Day Mortality After Elective On-Pump Coronary Artery Bypass Grafting
Source: Interdiscip Cardiovasc Thorac Surg. 2026 Jun 30;41(7):ivag190. doi: 10.1093/icvts/ivag190 (PMC13362241; doi:10.1093/icvts/ivag190)
Supplement: ivag190_Supplementary_Data [file ivag190_supplementary_data.zip › Supplementary Tables.docx]

**Supplementary Table 1. Full missingness matrix in the eligible cohort (n=492)**

| Pattern | Outcome | BNP | HALP | SII | PIV | COPD | ECA | CKD | DM | Prior CS | PAP≥40 | EF | NYHA | Creat | ESII | X-clamp | n | % |
| --- | --- | --- | --- | --- | --- | --- | --- | --- | --- | --- | --- | --- | --- | --- | --- | --- | --- | --- |
| 1 |  |  |  |  |  |  |  |  |  |  |  |  |  |  |  |  | 455 | 92.5 |
| 2 |  |  |  |  |  |  |  |  |  |  |  |  |  | ✓ |  |  | 11 | 2.2 |
| 3 | ✓ | ✓ | ✓ | ✓ | ✓ | ✓ | ✓ | ✓ | ✓ | ✓ | ✓ | ✓ | ✓ | ✓ | ✓ | ✓ | 11 | 2.2 |
| 4 | ✓ |  |  |  |  |  |  |  |  |  |  |  |  |  |  | ✓ | 1 | 0.2 |
| 5 | ✓ |  |  |  |  |  | ✓ | ✓ | ✓ | ✓ | ✓ |  | ✓ | ✓ | ✓ | ✓ | 1 | 0.2 |
| 6 | ✓ |  | ✓ | ✓ | ✓ |  | ✓ |  | ✓ |  |  |  |  |  |  | ✓ | 1 | 0.2 |
| 7 | ✓ |  | ✓ | ✓ | ✓ |  | ✓ |  | ✓ | ✓ |  | ✓ | ✓ | ✓ | ✓ | ✓ | 1 | 0.2 |
| 8 | ✓ |  | ✓ | ✓ | ✓ |  | ✓ | ✓ | ✓ | ✓ | ✓ | ✓ | ✓ | ✓ | ✓ | ✓ | 1 | 0.2 |
| 9 | ✓ |  | ✓ | ✓ | ✓ | ✓ |  | ✓ |  | ✓ |  | ✓ | ✓ | ✓ |  | ✓ | 1 | 0.2 |
| 10 | ✓ |  | ✓ | ✓ | ✓ | ✓ | ✓ | ✓ | ✓ |  | ✓ |  | ✓ |  | ✓ | ✓ | 1 | 0.2 |
| 11 | ✓ | ✓ |  |  |  |  |  | ✓ |  |  | ✓ |  |  | ✓ | ✓ | ✓ | 1 | 0.2 |
| 12 | ✓ | ✓ |  |  |  |  | ✓ |  |  |  |  | ✓ | ✓ | ✓ | ✓ | ✓ | 1 | 0.2 |
| 13 | ✓ | ✓ |  |  |  |  | ✓ |  |  | ✓ | ✓ | ✓ | ✓ | ✓ | ✓ | ✓ | 1 | 0.2 |
| 14 | ✓ | ✓ |  |  |  |  | ✓ | ✓ |  | ✓ | ✓ | ✓ | ✓ | ✓ | ✓ | ✓ | 1 | 0.2 |
| 15 | ✓ | ✓ |  |  |  |  | ✓ | ✓ | ✓ | ✓ | ✓ | ✓ | ✓ | ✓ | ✓ | ✓ | 1 | 0.2 |
| 16 | ✓ | ✓ |  |  |  |  | ✓ | ✓ | ✓ | ✓ | ✓ | ✓ | ✓ | ✓ | ✓ | ✓ | 1 | 0.2 |
| 17 | ✓ | ✓ | ✓ | ✓ |  |  | ✓ | ✓ |  |  | ✓ |  | ✓ | ✓ | ✓ | ✓ | 1 | 0.2 |
| 18 | ✓ | ✓ | ✓ | ✓ | ✓ |  | ✓ |  | ✓ | ✓ | ✓ | ✓ | ✓ | ✓ | ✓ | ✓ | 1 | 0.2 |

**Footnote:**✓ indicates missing data. Only variables with at least one missing value are shown. Variables without missingness in the eligible cohort were sex, age, CPB time, and graft configuration. Missingness was clustered rather than diffuse: 455 patients (92.5%) had complete data for all listed variables, 11 patients (2.2%) had isolated missing creatinine only, and the remaining 26 patients accounted for all missing 30-day outcome data and most additional missingness.

**Abbreviations:**BNP, B-type natriuretic peptide; CKD, chronic kidney disease; COPD, chronic obstructive pulmonary disease; Creat, serum creatinine; ECA, extracardiac arteriopathy; EF, ejection fraction; ESII, EuroSCORE II; HALP, hemoglobin, albumin, lymphocyte, and platelet score; NYHA, New York Heart Association; PAP≥40, pulmonary artery pressure ≥40 mmHg; PIV, pan-immune-inflammation value; Prior CS, previous cardiac surgery; SII, systemic immune-inflammation index; X-clamp, cross-clamp time.

**Supplementary Table 2. Missing-data pattern and reasons for exclusion in the eligible cohort (n=492)**

| Variable / data element | Missing, n (%) |
| --- | --- |
| 30-day mortality status | 26 (5.3) |
| BNP | 19 (3.9) |
| HALP | 18 (3.7) |
| SII | 18 (3.7) |
| PIV | 17 (3.5) |
| EuroSCORE II | 23 (4.7) |
| NYHA class | 23 (4.7) |
| Serum creatinine | 34 (6.9) |
| Cross-clamp time | 26 (5.3) |

**Footnote:**Missingness was clustered rather than isolated, and counts are therefore not additive. The primary analytic cohort was defined by complete 30-day mortality status and complete data for the prespecified primary predictors. Twenty-six otherwise eligible patients were excluded from the primary analysis because outcome and/or primary predictor data were unavailable. All 26 excluded patients had unavailable 30-day mortality status; most also had additional missing clinical or biomarker data. A full missingness matrix is provided in Supplementary Table 1.

**Abbreviations:**BNP, B-type natriuretic peptide; HALP, hemoglobin, albumin, lymphocyte, and platelet score; NYHA, New York Heart Association; PIV, pan-immune-inflammation value; SII, systemic immune-inflammation index.

**Supplementary Table 3. Univariable Firth penalized logistic regression models for 30-day mortality in the analytic cohort (n=466)**

| **Variable** | **Odds ratio (95% CI)** | **P value** |
| --- | --- | --- |
| EuroSCORE II (per 1-unit increase) | 1.87 (1.49–2.35) | <0.001 |
| HALP (per 5-unit decrease) | 2.42 (1.83–3.20) | <0.001 |
| log2BNP (per doubling) | 1.73 (1.41–2.12) | <0.001 |
| log2SII (per doubling) | 3.90 (2.07–7.33) | <0.001 |
| log2PIV (per doubling) | 2.49 (1.56–3.97) | <0.001 |

**Footnote:** Odds ratios were estimated using univariable Firth penalized logistic regression with 30-day mortality as the dependent variable. HALP was scaled per 5-unit decrease to improve clinical interpretability. BNP, SII, and PIV were log2-transformed; corresponding odds ratios therefore represent the relative increase in the odds of 30-day mortality per doubling of the biomarker value. P values are from penalized likelihood ratio tests.

**Abbreviations:**BNP, B-type natriuretic peptide; CI, confidence interval; HALP, hemoglobin, albumin, lymphocyte, and platelet score; PIV, pan-immune-inflammation value; SII, systemic immune-inflammation index.

**Supplementary Table 4. Inclusion-weighted sensitivity analysis for 30-day mortality in the analytic cohort**

| Variable | Model 1: IPW-adjusted OR (95% CI) | P value | Model 2: IPW-adjusted OR (95% CI) | P value |
| --- | --- | --- | --- | --- |
| EuroSCORE II (per 1-unit increase) | 1.81 (1.37–2.39) | <0.001 | 1.70 (1.31–2.20) | <0.001 |
| HALP (per 5-unit decrease) | 2.34 (1.76–3.11) | <0.001 | 2.54 (1.80–3.58) | <0.001 |
| log2BNP (per doubling) | — | — | 1.72 (1.31–2.26) | <0.001 |

**Footnote:**Sensitivity analyses were performed using stabilized inverse-probability-of-inclusion weighting (IPW). Inclusion probabilities were estimated in the full eligible cohort (n=492) using a logistic model with age, sex, cardiopulmonary bypass time, distal graft number, and LIMA use as predictors of inclusion in the primary analytic cohort. Weighted logistic regression models were then fitted in the analytic cohort (n=466) to assess whether the main associations were robust to potential selection bias arising from non-inclusion of otherwise eligible patients. HALP was scaled per 5-unit decrease. BNP was log2-transformed; the corresponding odds ratio therefore represents the change in the odds of 30-day mortality per doubling of BNP.

**Abbreviations:**BNP, B-type natriuretic peptide; CI, confidence interval; HALP, hemoglobin, albumin, lymphocyte, and platelet score; IPW, inverse-probability weighting; LIMA, left internal mammary artery; OR, odds ratio.

**Supplementary Table 5. Tipping-point analysis for unavailable 30-day outcomes among 26 excluded but otherwise eligible patients**

| Assumed deaths among excluded patients (n=26) | Excluded-group mortality, % | Total deaths in eligible cohort (n=492) | Overall eligible-cohort 30-day mortality, % | Excluded:included mortality ratio* |
| --- | --- | --- | --- | --- |
| 0 | 0.0 | 21 | 4.3 | 0.00 |
| 1 | 3.8 | 22 | 4.5 | 0.85 |
| 2 | 7.7 | 23 | 4.7 | 1.71 |
| 3 | 11.5 | 24 | 4.9 | 2.56 |
| 5 | 19.2 | 26 | 5.3 | 4.27 |
| 7 | 26.9 | 28 | 5.7 | 5.97 |
| 10 | 38.5 | 31 | 6.3 | 8.53 |
| 13 | 50.0 | 34 | 6.9 | 11.10 |

**Footnote:** The observed 30-day mortality in the primary analytic cohort was 4.5% (21/466). This tipping-point analysis examines how progressively more adverse assumptions regarding the 26 excluded patients with unavailable 30-day outcome data would affect the overall mortality estimate in the full eligible cohort. Because the excluded patients also had missing primary predictor data, these scenarios do not re-estimate the primary multivariable models; rather, they quantify the extent of outcome-related uncertainty introduced by the unavailable follow-up data.

* Calculated relative to the observed mortality in the included analytic cohort (21/466, 4.5%).

**Supplementary Table 6. Alternative multivariable Firth penalized logistic regression models including SII or PIV**

| Variable | Model A: EuroSCORE II + log2SII OR (95% CI) | P value | Model B: EuroSCORE II + log2PIV OR (95% CI) | P value | Model C: EuroSCORE II + HALP + log2SII OR (95% CI) | P value | Model D: EuroSCORE II + HALP + log2PIV OR (95% CI) | P value |
| --- | --- | --- | --- | --- | --- | --- | --- | --- |
| EuroSCORE II (per 1-unit increase) | 1.78 (1.41–2.25) | <0.001 | 1.81 (1.44–2.27) | <0.001 | 1.75 (1.34–2.29) | <0.001 | 1.73 (1.33–2.25) | <0.001 |
| HALP (per 5-unit decrease) | — | — | — | — | 2.46 (1.72–3.51) | <0.001 | 2.27 (1.65–3.14) | <0.001 |
| log2SII (per doubling) | 3.05 (1.52–6.12) | 0.002 | — | — | 0.62 (0.28–1.38) | 0.242 | — | — |
| log2PIV (per doubling) | — | — | 2.52 (1.41–4.48) | 0.002 | — | — | 0.84 (0.39–1.81) | 0.649 |

**Footnote:**Alternative multivariable models were fitted using Firth penalized logistic regression with 30-day mortality as the dependent variable. SII and PIV were evaluated in separate models to avoid overparameterization. In Models A and B, SII and PIV each showed significant associations with 30-day mortality beyond EuroSCORE II alone. However, after addition of HALP in Models C and D, neither SII nor PIV remained independently associated with mortality, whereas EuroSCORE II and HALP retained robust associations. Incremental penalized likelihood-ratio testing showed significant improvement for Model A versus EuroSCORE II alone (P<0.001) and for Model B versus EuroSCORE II alone (P<0.001), whereas addition of SII or PIV to Model 1 was not associated with significant incremental improvement (Model C vs Model 1, P=0.081; Model D vs Model 1, P=0.152).

**Abbreviations:**CI, confidence interval; EuroSCORE II, European System for Cardiac Operative Risk Evaluation II; HALP, hemoglobin, albumin, lymphocyte, and platelet score; OR, odds ratio; PIV, pan-immune-inflammation value; SII, systemic immune-inflammation index.

**Supplementary Table 7. Comparison of ridge-penalized and Firth-penalized primary models for 30-day mortality in the analytic cohort (n=466)**

| Variable / metric | Model 1 Firth OR (95% CI) | Model 1 Ridge OR* | Model 2 Firth OR (95% CI) | Model 2 Ridge OR* |
| --- | --- | --- | --- | --- |
| Variables |  |  |  |  |
| EuroSCORE II (per 1-unit increase) | 1.74 (1.33–2.27) | 1.70 | 1.61 (1.22–2.14) | 1.59 |
| HALP (per 5-unit decrease) | 2.23 (1.66–3.00) | 2.11 | 2.37 (1.67–3.37) | 2.18 |
| log2BNP (per doubling) | — | — | 1.69 (1.27–2.25) | 1.62 |
| Model performance |  |  |  |  |
| Apparent AUC | 0.976 | 0.975 | 0.980 | 0.980 |
| Brier score | 0.028 | 0.028 | 0.022 | 0.022 |

**Footnote:** Model 1 included EuroSCORE II and HALP. Model 2 included EuroSCORE II, HALP, and log2BNP. Firth estimates were obtained from penalized logistic regression using the Jeffreys prior penalty. Ridge estimates were obtained from L2-penalized logistic regression after predictor standardization, with the penalty selected by cross-validation; odds ratios were then back-transformed to the original predictor scales. HALP was scaled per 5-unit decrease. BNP was log2-transformed.

* Ridge models do not provide directly comparable profile-likelihood confidence intervals; therefore, point estimates are shown.

**Abbreviations:**AUC, area under the receiver operating characteristic curve; BNP, B-type natriuretic peptide; CI, confidence interval; HALP, hemoglobin, albumin, lymphocyte, and platelet score; OR, odds ratio.
