# Supplementary material for: Incremental Prognostic Value of the Haemoglobin-Albumin-Lymphocyte-Platelet Score and B-Type Natriuretic Peptide for 30-Day Mortality After Elective On-Pump Coronary Artery Bypass Grafting
Source: Interdiscip Cardiovasc Thorac Surg. 2026 Jun 30;41(7):ivag190. doi: 10.1093/icvts/ivag190 (PMC13362241; doi:10.1093/icvts/ivag190)
Supplement: ivag190_Supplementary_Data [file ivag190_supplementary_data.zip › Supplementary legend.docx]

**Supplementary Figure 1.** EuroSCORE II-adjusted spline plot for HALP. The curve shows the adjusted odds ratio for 30-day mortality across the observed range of HALP values, with the reference set at the median HALP value in the analytic cohort. Shaded areas indicate 95% bootstrap confidence intervals. Lower HALP values were associated with progressively higher adjusted odds of 30-day mortality.

**Supplementary Figure 2.** EuroSCORE II-adjusted spline plot for log2BNP. The curve shows the adjusted odds ratio for 30-day mortality across the observed range of log2-transformed BNP values, with the reference set at the median BNP value in the analytic cohort. Shaded areas indicate 95% bootstrap confidence intervals. Higher BNP values were associated with progressively higher adjusted odds of 30-day mortality.

**Supplementary Figure 3.** Decision curve analysis. Decision curves are shown for EuroSCORE II alone, Model 1 (EuroSCORE II + HALP), and Model 2 (EuroSCORE II + HALP + log2BNP), together with the “treat all” and “treat none” strategies. Across the displayed threshold range, both biomarker-augmented models provided greater net benefit than EuroSCORE II alone, and Model 2 showed the highest net benefit overall.
