# Supplementary figures and images for: Incremental Prognostic Value of the Haemoglobin-Albumin-Lymphocyte-Platelet Score and B-Type Natriuretic Peptide for 30-Day Mortality After Elective On-Pump Coronary Artery Bypass Grafting
Source: Interdiscip Cardiovasc Thorac Surg. 2026 Jun 30;41(7):ivag190. doi: 10.1093/icvts/ivag190 (PMC13362241; doi:10.1093/icvts/ivag190)

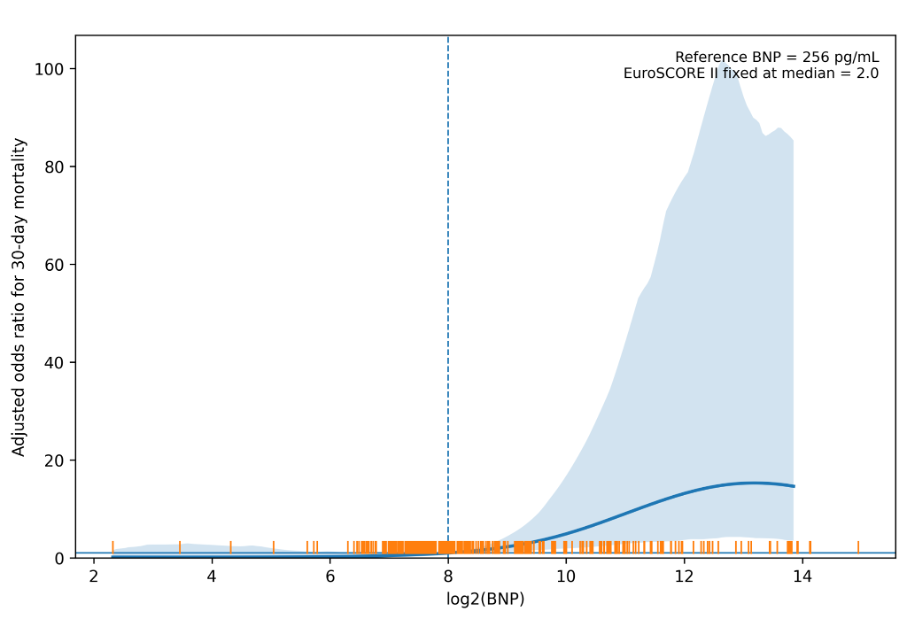

Supplement: ivag190_Supplementary_Data [file ivag190_supplementary_data.zip › S figure 2.tiff]

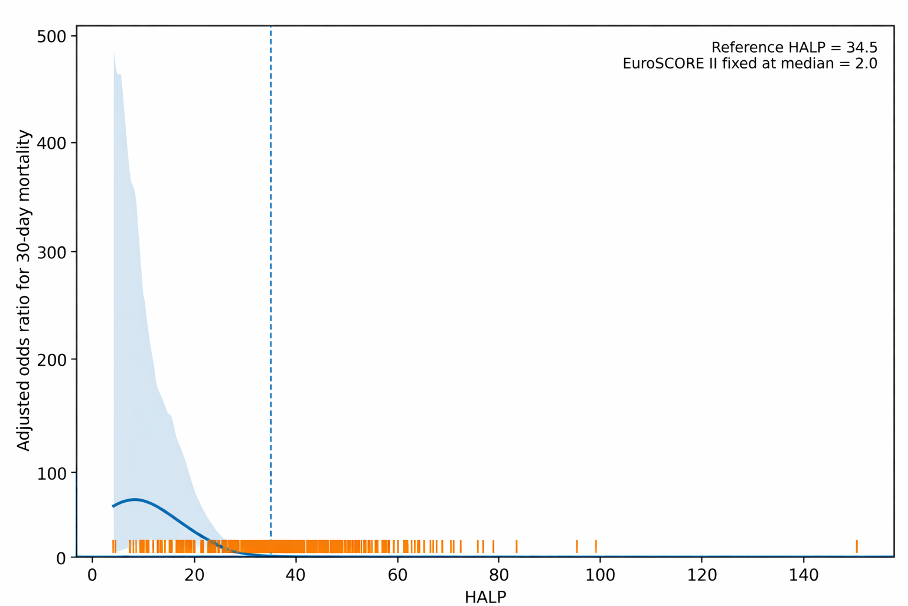

Supplement: ivag190_Supplementary_Data [file ivag190_supplementary_data.zip › S figure 1.tiff]

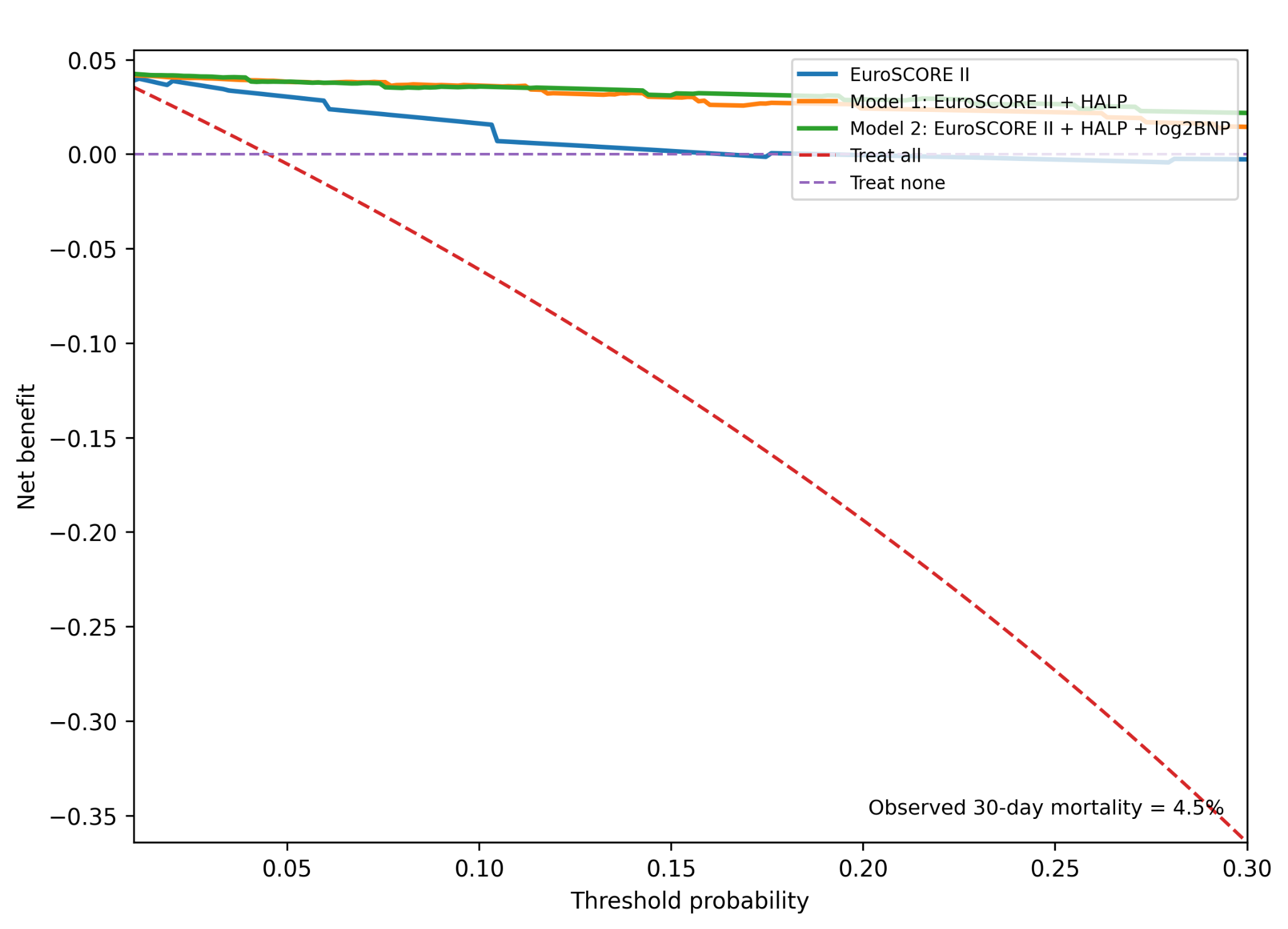

Supplement: ivag190_Supplementary_Data [file ivag190_supplementary_data.zip › S figure 3.tiff]
